# Fine Motor Skills, Executive Function, and School Readiness in Preschoolers with Externalizing Behavior Problems

**DOI:** 10.3390/bs15050708

**Published:** 2025-05-21

**Authors:** Atefeh Karimi, Bridget Poznanski, Katie C. Hart, Eliza L. Nelson

**Affiliations:** 1 Department of Psychology, Florida International University, Miami, FL 33199, USA; khart@fiu.edu (K.C.H.); elnelson@fiu.edu (E.L.N.); 2 Department of Pediatrics, Chobanian & Avedisian School of Medicine, Boston University, Boston, MA 02118, USA; bridget.poznanski@bmc.org; 3 Center for Children and Families, Florida International University, Miami, FL 33199, USA

**Keywords:** fine motor skills, executive function, school readiness, externalizing behavior, preschoolers

## Abstract

The objective of this study was to examine whether fine motor skills (FMS) and executive function (EF) are unique predictors of school readiness (SR). The sample was 108 preschoolers with externalizing behavior problems (EBP; Mean ± SD = 60.37 ± 3.94 months pre-intervention, 68% male) enrolled in a comprehensive 7-week school readiness summer program open trial. FMS were measured with the Learning Accomplishment Profile Diagnostic Third Edition (LAP-D); EF was measured with the Head–Toes–Knees–Shoulders (HTKS), and SR was measured with the Bracken School Readiness Assessment Third Edition (BSRA-3). All assessments were given pre- and post-intervention. All models controlled for participant age and socio-economic status (SES). Examining data pre-intervention, FMS but not EF uniquely predicted SR, explaining 46% of the variance. At post-intervention, both FMS and EF predicted SR, explaining 33% of the variance. These findings underscore the importance of screening both FMS and EF in preschoolers with EBP as they prepare to transition to kindergarten, as these domains both contribute to characterizing SR.

## 1. Introduction

Fine motor skills and executive function have been linked to school readiness skills during the preschool years (M. M. McClelland & Cameron, 2019). Although often studied and modeled separately in relation to school readiness, research suggests that fine motor skills and executive function, while correlated, independently contribute to early academic achievement (Malone et al., 2022). Yet, the role each domain plays in school readiness needs to be disentangled (Cameron et al., 2012). Preschoolers with externalizing behavior problems (EBP) who show a range of disruptive behaviors such as aggression, defiance, inattention, and hyperactivity/impulsivity have been a population of interest in school readiness studies, as they experience difficulties transitioning to kindergarten (Hart et al., 2019). While studies have reported that preschoolers with EBP have deficits in their fine motor skills (King-Dowling et al., 2015; Pagani & Messier, 2012; Peyre et al., 2019) as well as deficits in executive function (Schoemaker et al., 2013), the extent to which these skills contribute to school readiness in this population is unknown. This study addresses this knowledge gap by examining fine motor skills, executive function, and school readiness in preschoolers with EBP.

### 1.1. School Readiness and Fine Motor Skills

School readiness refers to a child’s level of preparedness to enter formal education, and it is measured by various skills (Mashburn & Pianta, 2006). The contemporary literature highlights that school readiness goes beyond traditional academic domains like numeracy and reading to include physical, behavioral, social–emotional, and attentional competencies that together contribute to future academic achievement (Blair, 2002; Duncan et al., 2007; Pan et al., 2019). Among the various competencies contributing to school readiness, motor skills have recently received attention in the literature (e.g., Kamphorst et al., 2021). Motor skills are divided into two types: gross motor skills, which involve the use of large muscles for movements such as crawling, sitting, and walking, and fine motor skills, which involve the use of smaller muscles of hands and wrists for movements such as manipulation, drawing, writing, and grasping (Gonzalez et al., 2019).

Extensive research has demonstrated a link between fine motor skills and school readiness skills (for a review, see M. M. McClelland & Cameron, 2019). The link between fine motor skills and school readiness is evident as early as the preschool years. For instance, Ricciardi et al. (2021) showed that fine motor skills at 4 years of age predicted academic performance at kindergarten as well as in the first, third, and fifth grades. Moreover, a study with 4- to 5-year-old children found that fine motor skills were a concurrent predictor of school readiness (Jones et al., 2021). Likewise, Dinehart and Manfra (2013) studied two types of fine motor skills, manipulation and writing, as predictors of later academic performance. Results found that both manipulation and writing at 5 years were predictive of second-grade math and reading, although writing was the stronger predictor of the two. There is also an association between fine motor skills and school readiness during kindergarten. Grissmer et al. (2010) showed that fine motor skills measured at kindergarten entry strongly predicted reading and math skills in fifth grade.

### 1.2. School Readiness and Executive Function

Executive function is a higher-order cognitive construct that integrates components such as cognitive flexibility, working memory, and inhibitory control utilized in planning and performing goal-directed activities, problem-solving, and self-regulation (Blair, 2002; Zelazo, 2015). Executive function is essential for self-regulation, an important skill for school readiness, which allows children to manage their emotions, sustain attention, and engage effectively in preschool tasks (Blair & Raver, 2015; Cameron et al., 2012). In contrast to activities that require an automatic response, executive function involves active manipulation of information (Nayfeld et al., 2013). Preschool activities require attending to instructions and holding them in memory, manipulating information, and inhibiting responses to distracting stimuli—all of which require components of executive function (Welsh et al., 2010). As a result, executive function has been studied as a potential predictor of school readiness skills in the literature (Mann et al., 2017).

A large number of studies have reported that advances in preschoolers’ executive function are linked to children’s school readiness skills and, later, academic success (Bull et al., 2011; Shaul & Schwartz, 2014). For example, executive function in 3- to 4-year-old children was predictive of prekindergarten math, vocabulary, and literacy abilities (M. M. McClelland et al., 2007). Moreover, Blair and Razza (2007) found that executive function in 3- to 5-year-old children predicted math and literacy ability in kindergarten. Not only has executive function been predictive of academic school readiness skills, but associations between executive function and non-academic school readiness skills have also been found. For example, advances in executive function in the preschool years were positively associated with social competence (Razza & Blair, 2009). Finally, preschoolers with better executive function at age 3 had fewer problem behaviors at age 4 (Hughes & Ensor, 2008).

### 1.3. School Readiness, Fine Motor Skills, and Executive Function

The relationship between fine motor skills and executive function to school readiness is complex; various investigators have been interested in whether these domains uniquely predict school readiness or, alternatively, whether they share variance in explaining school readiness outcomes (Cameron et al., 2012). In one study, executive function and fine motor skills in 3- to 5-year-old children uniquely and distinctively predicted school readiness skills at the beginning of the kindergarten year. Executive function was strongly associated with math and literacy outcomes, while fine motor skills, especially copying shapes and designs, were strongly associated with the letter–word identification and sound awareness (Cameron et al., 2012). Another study found that fine motor skills were significantly predictive of school readiness skills when executive function was also considered in 4- to 5-year-old children, suggesting that fine motor skills play a more crucial role than executive function in school readiness (Aydoner & Bumin, 2023). These studies suggest fine motor skills and executive function may uniquely predict school readiness outcomes.

However, in some studies, fine motor skills and executive function shared variance in predicting school readiness. For instance, in a study that followed 3-year-old preschoolers to the first grade, fine motor skills and executive functions both contributed to the same profile of school readiness after a latent class analysis was conducted (Kamphorst et al., 2021). In another longitudinal study, the association between fine motor skills at age 5 and math and reading skills at age 6 was fully accounted for by executive function, negating fine motor skills and executive function as unique predictors of academic achievement (Malone et al., 2022). Together, these studies argue that executive function and fine motor skills might alternatively share some variance in predicting school readiness outcomes.

Considering the mixed evidence from prior studies, with some supporting unique contributions of executive function and fine motor skills and some supporting shared variance between them, further examination is needed to disentangle the complexities of these links. The present study aimed to build on this growing body of work by examining the extent to which fine motor skills and executive function predict school readiness in preschoolers with EBP.

### 1.4. Fine Motor Skills and Executive Function in EBP

Previous studies have linked EBP and fine motor skill deficits, as well as EBP and executive function deficits, but we are unaware of any study examining these variables together. Children with EBP often exhibit deficits in their fine motor skills (Livesey et al., 2006). For instance, 3-to-6-year-old children at risk for motor difficulties have higher aggression and EBP scores (King-Dowling et al., 2015), while inattention and hyperactivity in kindergarteners have been linked to lower fine motor skills (Pagani & Messier, 2012). Additionally, Peters et al. (2014) found that motor deficits co-occurred with externalizing behaviors, such as aggression and attention problems, in school-age children. Longitudinally, Peyre et al. (2019) reported that inattention symptoms in preschool years were associated with lower fine motor scores in kindergarten and first grade. Likewise, children with EBP often exhibit executive function deficits (Willcutt et al., 2005). Meta-analyses have shown that EBP symptoms, including aggression, conduct problems, and oppositionality, have been linked with lower executive function skills (Morgan & Lilienfeld, 2000; Schoemaker et al., 2013).

### 1.5. Current Study

The current study assessed two different types of fine motor skills, manipulation and writing, as well as executive function, alongside school readiness skills prior to and after a 7-week comprehensive summer program aimed at improving the school readiness of preschoolers with EBP for the kindergarten transition (for full details of the intervention see Hart et al., 2024). This summer school readiness program was an open trial for children living in urban poverty. All children received services as required by the local agency that supported this program. Therefore, there were no control groups in this study (i.e., children with EBP who did not receive the intervention and children without EBP). We tested fine motor skills and executive function as predictors of school readiness before and after the intervention. To the best of our knowledge, it is the first study examining these relations in a sample of preschoolers with externalizing behavior problems; therefore, we did not have specific hypotheses for each time point. We believed it was important to capture any changes across this program between these variables as a first step.

We expected fine motor skills would be associated with school readiness, such that preschoolers with EBP with more proficient fine motor skills in both domains would exhibit higher school readiness skills. Moreover, we expected that preschoolers with EBP who have more advanced executive function would show higher school readiness skills. We predicted that fine motor skills and executive function would uniquely predict school readiness skills in children with EBP. To test our hypotheses, we used regression analysis. We first examined three single-domain models with fine motor manipulation, fine motor writing, or executive function as the predictor and school readiness as the outcome, and then one model with fine motor skills and executive function together as predictors and school readiness as the outcome for comparison. All models were run separately for pre- and post-intervention data to examine if the relations between our variables of interest changed after intervention.

## 2. Materials and Methods

### 2.1. Participants

The participants in this study were 128 preschoolers enrolled in a 7-week summer school readiness program designed for rising kindergarteners with EBP (for details on the open trial, see Hart et al., 2024). The goal of this program was to improve school readiness by addressing behavioral, social–emotional, and academic skills through evidence-based practice. Data were collected at both baseline and post-intervention to examine how fine motor skills and executive functions related to school readiness in this population. While fine motor skills were not an inclusion criterion for this program or the primary focus of the intervention, they were considered a secondary focus of the intervention and were assessed as part of comprehensive program evaluations. This study was conducted in a large urban area in the Southeastern United States. Participants came from historically marginalized, underserved, and low-income backgrounds. Recruitment was carried out through local preschools, mental health agencies, brochures, social media advertisements, e-newsletters, and school-based workshops. Families interested in this study were invited to a screening appointment.

To be eligible, children had to (1) transition to kindergarten in the upcoming school year; (2) exhibit behavioral, learning, attention, or social–emotional challenges as confirmed by a diagnosis of a behavioral or emotional disorder (e.g., Attention-Deficit/Hyperactivity Disorder, Oppositional Defiant Disorder) or qualification for special education services through an Individualized Education Program (IEP); (3) possess sufficient cognitive and language abilities to engage in this program; and (4) be able to attend the 7-week intervention as well as weekly parent training. Enrollment decisions were made based on clinical judgment, assessment scores, caregiver and teacher reports, and direct observations rather than a single diagnostic criterion or score serving as the sole exclusion criterion.

**Figure 1:**
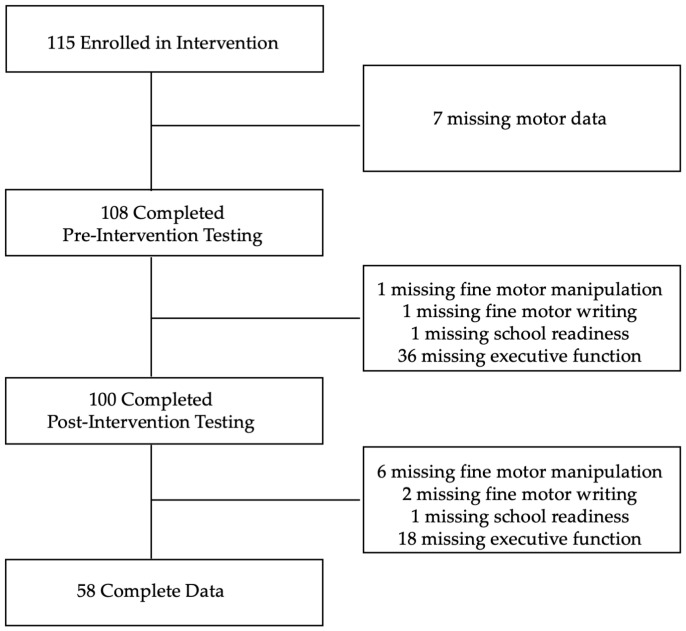
Consort Diagram.

Demographic data for children and parents are presented in Table 1 and Table 2, respectively. The sample was predominantly Black (92.59%), with approximately 87.98% of families living at or below the poverty line. Data were collected across three cohorts between 2017 and 2019 and then combined for the analysis (Figure 1). At pre-intervention, preschoolers were 52 to 70 months old (Mean ± SD = 60.37 ± 3.94 months, 68.52% male). At post-intervention, preschoolers were 58 to 71 months old (Mean ± SD = 64.83 ± 3.72 months, 68.52% male).

### 2.2. Measures

#### 2.2.1. Learning Accomplishment Profile Diagnostic 3rd Edition (LAP-D)

The Learning Accomplishment Profile-Diagnostic (LAP-D) is a standardized, norm-referenced assessment designed for children aged 30 to 72 months. It evaluates four domains: fine motor, cognitive, language, and gross motor skills (Nehring & Nering, 1992). This study focused on the fine motor domain, which is divided into two subscales: fine motor manipulation (FM) and fine motor writing (FW). The FM subscale includes 28 items that assess children’s manual dexterity through tasks involving object manipulation (e.g., building towers with blocks, turning book pages, and cutting with scissors). The FW subscale consists of 31 items measuring early writing skills through activities such as copying letters and drawing basic shapes. The LAP-D demonstrates strong psychometric properties, including high internal consistency (FM α = 0.91, FW α = 0.96), strong test–retest reliability (FM r = 0.91, FW r = 0.96), and robust construct and criterion validity (Hardin et al., 2005). In this study, raw LAP-D scores were used in the regression analyses. Raw LAP-D scores range from 0 to 28 for the FM subscale and from 0 to 31 for the FW subscale.

#### 2.2.2. Bracken School Readiness Assessment 3rd Edition (BSRA-3)

The Bracken School Readiness Assessment, Third Edition (BSRA-3), is a widely used measure of school readiness, comprising five subtests: colors, letters, numbers/counting, size/comparison, and shapes (Bracken, 2007). Crafted to assess concept acquisition and receptive language skills, it is appropriate for children between the ages of 2.5 and 7 years. The BSRA-3 demonstrates strong psychometric properties, with an overall internal consistency of 0.98 and reliability coefficients ranging from 0.96 to 0.99 across age groups (Panter & Bracken, 2009). Each subtest also exhibits high internal consistency, with reliability values ranging from 0.85 to 0.98 (Panter & Bracken, 2009). In terms of concurrent validity, the BSRA-3 has shown moderate to high correlations with the Peabody Picture Vocabulary Test-III (r = 0.69 to 0.79). Additionally, it has been validated as a strong predictor of children’s academic success, showing its value in assessing kindergarten readiness (Panter & Bracken, 2009). The raw scores for BSRA-3 range from 0 to 88.

#### 2.2.3. Head–Toes–Knees–Shoulders (HTKS)

Executive function was assessed using the Head–Toes–Knees–Shoulders (HTKS) task (Ponitz et al., 2009), a widely used measure of executive functioning in preschool-aged children. A meta-analysis has shown a positive association between performance on HTKS and children’s academic performance (Kenny et al., 2023). The HTKS requires children to perform behavioral responses in the opposite way of verbal instructions (e.g., when instructed to “touch your head,” they must instead touch their toes). This task has been designed to assess behavioral self-regulation, which involves components of executive function such as controlling attention, working memory, and inhibitory control, all of which contribute to school readiness (Cameron et al., 2012). The HTKS has demonstrated strong psychometric properties (Ponitz et al., 2009; Wanless et al., 2011) and has been validated in preschoolers with externalizing behavior problems (Graziano et al., 2015). HTKS scores range from 0 to 60, with 2 points awarded for correct initial responses, 1 point for self-corrected responses, and 0 points for incorrect responses. Higher scores indicate stronger executive functioning abilities. If the child makes four consecutive errors in one section, the test is discontinued.

#### 2.2.4. Behavior Assessment System for Children 3rd Edition (BASC-3)

The Behavior Assessment System for Children, Third Edition (BASC-3), is a norm-referenced questionnaire designed to evaluate children’s behavioral and emotional functioning (Reynolds et al., 2015). In this study, the parent-report form for preschool-aged children, consisting of 139 items, was administered. The BASC-3 includes nine clinical scales—Hyperactivity, Aggression, Anxiety, Depression, Somatization, Atypicality, Withdrawal, Attention Problems, and Developmental Social Disorders—as well as five adaptive scales—Adaptability, Social Skills, Functional Communication, Activities of Daily Living, and Leadership. Parents rate the frequency of behaviors observed over the past six months on a four-point scale (never, sometimes, often, almost always). The assessment takes approximately 10 to 20 min to complete. BASC-3 shows appropriate internal consistency (Cronbach’s Alpha = ranging from 0.75 to 0.90 in different scales) (Reynolds et al., 2015) and test–retest reliability with coefficients between 0.70 and 0.90 (Reynolds et al., 2015). This measure also shows strong validity. BASC-3 scores were converted to T-scores, with a mean of 50 and a standard deviation of 10. Scores on the Attention Problems, Hyperactivity, and Aggression subscales were used in analyses.

### 2.3. Procedure

The study protocol was approved by the Florida International University Institutional Review Board (IRB-16-0101). Families first completed an initial screening process, starting with a brief phone call where caregivers provided basic information about their child and completed BASC-3. Children were invited for an in-person visit if caregivers reported elevated scores (60 or above) on the Attention Problems, Hyperactivity, or Aggression subscales of the BASC-3, if the child had an Individualized Education Program (IEP) at school, or if caregivers expressed clinically significant concerns. Parents provided written informed consent for their child’s participation. The in-person assessment included two stages: an initial eligibility visit (Visit 1) and an enrollment visit (Visit 2) for those who met the eligibility criteria. The BASC-3 was completed by parents over the phone as part of the pre-intervention assessment and again at the end of the intervention. Direct child assessments, including the BSRA-3 and the HTKS task, were conducted before and after the intervention. The LAP-D was administered during the first and last weeks of this program. Although there was no specific activity aimed at improving fine motor skills in this program, the curriculum included tasks that required fine motor manipulation and fine motor skills. For instance, children used manipulation skills in activities such as turning pages, inserting objects into and removing objects from one another, stabilizing paper, removing lids, and holding utensils. They also used writing skills for tracing, scribbling, and circling while doing their science and math activities.

All assessments took place in an early childhood center in a local community where this summer school readiness program was held. Children were randomly signed out of their classrooms for the LAP-D assessment, which was administered individually in a quiet room by trained assessors. Children received a small toy after completing each LAP-D fine motor subscale. The order of tests was randomized, and all assessments were conducted between 9:30 a.m. and 2:30 p.m.

### 2.4. Statistical Analysis

A paired sample *t*-test was used to examine the differences between fine motor skills, executive function, school readiness, and externalizing behavior pre- and post-intervention. These tests were conducted to aid in the interpretation of the regression models, as we did not know a priori if the relations between our target variables would change from pre- to post-intervention. Linear regression analysis was used to predict school readiness outcomes from fine motor skills and executive function at both pre- and post-intervention timepoints. For each timepoint, we first examined separate models with FM, FW, or HTKS as predictors and total BSRA-3 scores as the outcome, and then one model with FM, FW, and HTKS altogether as predictors and total BSRA-3 as the outcome. Models were tested separately for pre- and post-intervention data. This approach allowed us to tease apart the effects of fine motor and executive function skills on school readiness. LAP-D, HTKS, and BSRA-3 raw scores were used in regression models. Parents’ income and participants’ education and age were controlled in all models. All statistical analyses were conducted in RStudio (v. 4.3.2.) with an alpha level of 0.05. In each statistical test, individuals were excluded if they were missing data for one of the variables in the analysis using listwise deletion.

## 3. Results

### Descriptive Statistics

Descriptive statistics for all the assessments in this study pre- and post-intervention can be seen in Table 3 and Table 4, respectively. Skewness values for all the variables ranged from −1.43 to 1.35, while kurtosis values ranged from −1.23 to 2.62, indicating that the distribution approximates normality, supporting the use of parametric tests. Results from the paired *t*-tests indicated that there was a significant reduction in externalizing symptoms post-intervention (*t*(99) = 6.55, *p* < 0.001, *d* = 0.64, CI = 6.03, 11.28). Fine motor manipulation skills improved significantly post-intervention (*t*(100) = 4.82, *p* < 0.001, d = 0.36, CI = 0.65, 1.56), and a similar effect was seen for fine motor writing scores (*t*(98) = 5.91, *p* < 0.001, *d* = 0.35, CI = 1.28, 2.58). Executive function also improved significantly post intervention *(t*(64) = 4.81, *p* < 0.001, *d* = 0.62, CI = 5.65, 13.67). Lastly, school readiness skills improved from pre to post-intervention (*t*(105) = 17.24, *p* < 0.001, *d* = 0.81, CI = 12.49, 15.74).

Table 5 shows the estimates of the four pre-intervention regression model coefficients along with their corresponding standard errors, 95% confidence intervals, and *p*-values. In the model with HTKS scores as the predictor and total BSRA-3 score as the outcome, there was a significant effect of HTKS scores on BSRA-3 scores ${(R}^{2}$ = 0.12, *F*(4, 62) = 2.30, *p* < 0.01). For every one-unit increase in HTKS scores, there was an associated increase of 0.41 units in BSRA-3 scores. Parent education, income, and participant age were not significant predictors. Overall, this model explained 12% of the variance in total school readiness. Likewise, in the model with FM scores as the predictor and total BSRA-3 score as the outcome, there was a significant effect of FM scores on BSRA-3 scores ${(R}^{2}$ = 0.21, *F*(4, 95) = 6.34, *p* < 0.001). For every one-unit increase in FM scores, there was an associated increase of 2.39 units in BSRA-3 scores. Parent education, income, and participant age were not significant predictors. Overall, this model explained 21% of the variance in total school readiness. Similarly, in the model with FW scores as the predictor and total BSRA-3 score as the outcome, there was a significant effect of FW scores on BSRA-3 scores ${(R}^{2}$ = 0.40, *F*(4, 25) = 16.28, *p* < 0.001). For every one-unit increase in FW scores, there was an associated increase of 2.00 units in BSRA-3 scores. Parent education, income, and participant’s age were not significant predictors. Overall, this model explained 40% of the variance in total school readiness.

**Figure 2:**
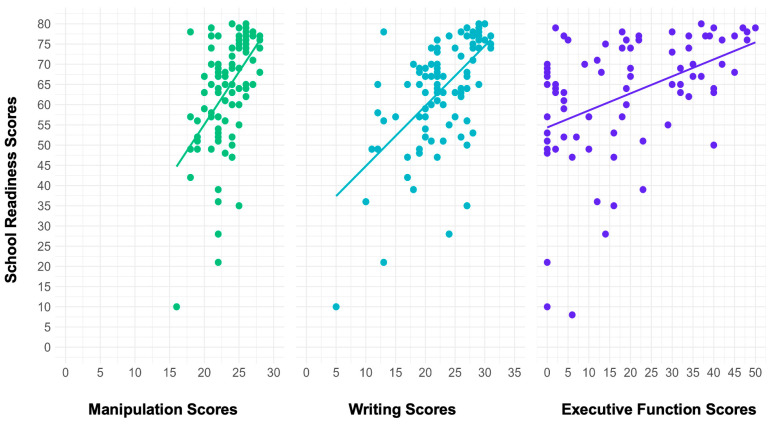
Scatter plots with regression lines showing the relation between school readiness scores and the three predictors pre-intervention.

In the model with FM, FW, and HTKS as the predictors and total BSRA-3 score as the outcome, there was a significant effect of only FW scores on BSRA-3 scores ${(R}^{2}$ = 0.46, *F*(6, 59) = 8.25, *p* < 0.001). For every one-unit increase in FW scores, there was a corresponding increase of 1.77 units in BSRA-3 scores. FM scores, HTKS scores, parent education, and participant age were not significant predictors; however, parents’ income was. For every one-unit increase in income, there was an associated decrease of 1.67 in BSRA-3 scores. Overall, this model, with manipulation and writing as predictors, explained 46% of the variance in total school readiness (Figure 2).

Table 6 shows the estimates of the four post-intervention regression model coefficients along with their corresponding standard errors, 95% confidence intervals, and *p*-values. In the model with HTKS scores as the predictor and total BSRA-3 score as the outcome, there was a significant effect of HTKS scores on BSRA-3 scores ${(R}^{2}$ = 0.25, *F*(4, 78) = 6.38, *p* < 0.001). For every one-unit increase in HTKS scores, there was an associated increase of 0.35 units in BSRA-3 scores. Parents’ education, income, and participant’s age were not significant predictors. Overall, this model explained 25% of the variance in total school readiness. Likewise, in the model with FM scores as the predictor and total BSRA-3 score as the outcome, there was a significant effect of FM scores on BSRA-3 scores ${(R}^{2}$ = 0.19, *F*(4, 91) = 6.04, *p* < 0.001). For every one-unit increase in FM scores, there was an associated increase of 2.10 units in BSRA-3 scores. Parents’ education, income, and participant’s age were not significant predictors. Overall, this model explained 21% of the variance in total school readiness. Similarly, in the model with FW scores as the predictor and total BSRA-3 score as the outcome, there was a significant effect of FW scores on BSRA-3 scores ${(R}^{2}$ = 0.30, *F*(4, 90) = 9.68, *p* < 0.001). For every one-unit increase in FW scores, there was an associated increase of 1.39 units in BSRA-3 scores. Parents’ education, income, and participant’s age were not significant predictors. Overall, this model explained 30% of the variance in total school readiness.

**Figure 3:**
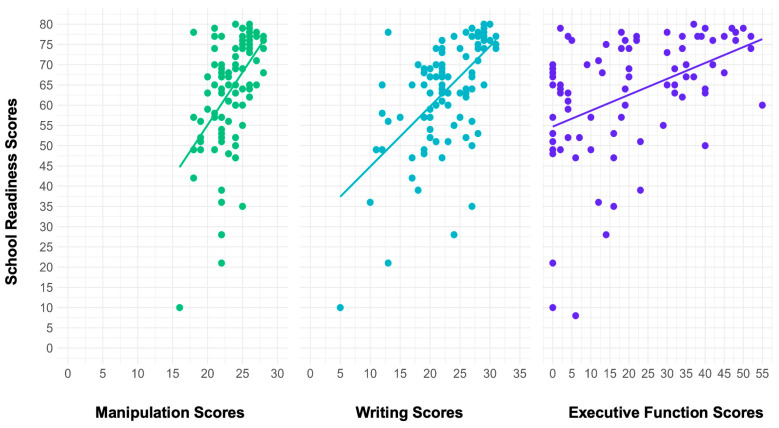
Scatter plots with regression lines showing the relation between school readiness scores and the three predictors post-intervention.

In the model with FM, FW, and HTKS as the predictors and total BSRA-3 score as the outcome, there was a significant effect of FM scores and HTKS scores on BSRA-3 scores ${(R}^{2}$ = 0.29, *F*(6, 70) = 5.71, *p* < 0.05). For every one-unit increase in HTKS scores, there was an associated increase of 0.19 units in BSRA-3 scores. For every one-unit increase in FW scores, there was an associated increase of 0.99 units in BSRA-3 scores. FM scores, parents’ education and income, and participant’s age were not significant predictors. Overall, this model explained 33% of the variance in total school readiness (Figure 3).

## 4. Discussion

The goal of the current study was to examine whether fine motor skills and executive function uniquely predict school readiness in preschoolers with EBP. Our results indicated that after attending the summer intervention, fine motor skills, executive function, and school readiness skills improved in this sample. In the models with fine motor skills and executive function as separate predictors, both domains significantly predicted school readiness outcomes. However, when predictors were examined together, fine motor skills emerged as a unique predictor pre-intervention, and both fine motor skills and executive function emerged as significant predictors post-intervention. Statistically, these findings mean that prior to intervention, executive function did not predict something unique and different from fine motor skills and the other predictors in the combined model. After intervention, fine motor writing, but not manipulation and executive function, were unique predictors for school readiness. We consider the results from the single-predictor models first before explaining the combined predictor models.

Results from the single-predictor models confirmed the predicted link between fine motor skills and school readiness in children with EBP. This pattern was in harmony with findings in the literature on children without EBP (Grissmer et al., 2010; Pagani & Messier, 2012; Ricciardi et al., 2021). Preschool tasks involve both cognitive and motor demands (Cameron et al., 2012). Preschoolers’ ability to develop automaticity in motor-related tasks such as manipulation and writing may influence the amount of cognitive capacity they can allocate to other learning objectives. On the contrary, children who struggle with the motor demands of preschool tasks, such as holding a pencil, will have less cognitive capacity to process other aspects of the task (Cameron et al., 2016; Medwell et al., 2007). Findings from the single-predictor models using executive function also found predictive links to school readiness in children with EBP, which is in line with prior studies on preschoolers without EBP and with our predictions (Blair & Razza, 2007; M. M. McClelland et al., 2007; Shaul & Schwartz, 2014). Preschool tasks require problem-solving, recalling, maintaining, and manipulating information in novel ways, following instructions, and sustaining attention (Cameron et al., 2012). Examined this way, both domains were predictors for school readiness in children with EBP. However, we also observed small to medium correlations between fine motor skills and executive function, prompting us to examine all predictors in combined models to determine which, if any, were unique predictors for school readiness pre- and post-intervention.

In the models where all predictors were considered, fine motor skills were the unique predictor of school readiness skills in children with EBP pre-intervention, and both executive function and fine motor skills were predictors of school readiness skills in children with EBP post-intervention. This change in predictors may be explained by considering that the primary goal of the intervention was to address the behavioral skills in participants, which may more closely align with executive function rather than fine motor skills. It is possible that executive function skills may moderate the impact of the intervention, though this hypothesis needs further investigation, as it was not the goal of the current study. Alternatively, greater fine motor skills may compensate for poorer executive function or vice versa. In a study by Cameron et al. (2015), visual motor integration, inhibitory control, and academic knowledge were tested at the beginning and the end of the preschool year in a sample of 467 children. Multilevel regression models found that children with advanced visuomotor integration but low inhibitory control performed similarly to those with high inhibitory control but poor visuomotor integration, and vice versa, at both timepoints. Moreover, a longitudinal study on children with motor delays found that those with more advanced fine motor skills at 10 to 14 months and 20 to 25 months exhibited higher executive function at 36 months (Cunha et al., 2024). These findings suggest that there may be more complex relations between fine motor skills, executive function, and school readiness. A dynamic link between these variables, creating a compensatory mechanism, may explain the shift in predictors from pre- to post-intervention in our dataset for children with EBP. However, a larger sample size is required to test this potential mechanism in future work. Similarly, EF skills may have moderated the impact of the intervention, though this needs future investigation.

We observed that the variance explained for school readiness dropped in our models from pre- to post-intervention. This difference may be due to the comprehensiveness of this program and the overall goal of improving school readiness. Indeed, children showed significant improvements in all three areas: fine motor skills (manipulation and writing), executive function, and school readiness. It is likely that there was greater variability in scores pre-intervention relative to post-intervention. A reduction in individual differences may have decreased the predictive power of the post-intervention model. Alternatively, we cannot rule out the contribution of potential predictors that we did not examine in this study, such as the quality of parent–child interactions and language skills (Linder et al., 2013; Prior et al., 2011), as well as mastery motivation (Amukune & Józsa, 2023).

### Limitations and Future Directions

While this study provided valuable insights into fine motor skills and executive function as predictors of school readiness in preschoolers with EBP, the generalizability of its findings may be limited. The local agency that supported the intervention required that all children receive services; therefore, there was no control group of children with EBP that followed longitudinally and did not participate in the school readiness intervention. Moreover, the sample was predominantly black and low-income, which is not representative of all children with EBP. We want to stress that these limitations for generalizability are also the unique aspects of this study in its diverse sample, which broadens behavioral science (Henrich et al., 2010).

In addition to the sample, there were also limitations with the measurements used in this study. To perform HTKS items, some proficiency in motor skills was required. The reliance on motor skills for the HTKS could also explain why HTKS scores in this study were clustered toward the bottom. To minimize the confounding effect of motor skills on the HTKS, a revised version called the HTKS-R has been developed (M. M. McClelland et al., 2021). The revised version includes items that ask children to respond verbally to prompts, e.g., “When I say toes, you say head”, with the purpose of reducing the motor demands of the task. This version was not available for the cohorts reported here (2017–2019). We have started utilizing the HTKS-R in data collection, but we do not yet have sufficient data for analyses. For performing LAP-D items, preschoolers needed to follow instructions, which required executive function. To mitigate executive function demands, we administered the manipulation items separately from the writing items with a break in between, and sometimes on different days, depending on the child.

To advance research on the link between fine motor skills and executive function, the application of more precise methodologies may be required. Cassidy and Willoughby (2024) proposed researchers should go beyond correlational studies and separate behavioral assessments of fine motor skills and executive function. They argue researchers should use corticomuscular coherence (CMC) instead. CMC measures the functional connectivity between the motor cortex and muscles during the motor tasks using EEG and EMG. CMC provides a more integrated picture of motor-executive function interactions by capturing brain–muscle synchronization. Since complex motor tasks involve executive function components like attention, planning, and inhibition, CMC can serve as a biomarker of typical motor development and track developmental changes more effectively than behavioral measures alone (V. M. McClelland et al., 2020). Additionally, CMC can be used to assess the effectiveness of motor interventions, revealing whether improvements in motor control and motor–cognition integration also enhance executive function development.

## 5. Conclusions

Identifying fine motor skills and executive function as unique predictors of school readiness in preschoolers with EBP carries important clinical and educational implications. Our findings demonstrate that fine motor skills account for a substantial portion of school readiness, while executive function also emerges as a key contributor to school readiness. These findings contribute to the existing literature by supporting the unique role of fine motor skills and executive function in predicting school readiness in children with EBP. Our findings also highlight the importance of screening both fine motor skills and executive function as markers of school readiness skills in EBP.

## Figures and Tables

**Table 1:** Demographics for children.

Characteristic	Percentage in Sample	n
Child’s sex		
Male	68.52%	74
Female	31.48%	34
Child’s race		
Black or African American	92.59%	100
White	2.78%	3
Multiracial	0.93%	1
Asian	0.93%	1
Other	2.78%	3
Child’s ethnicity		
Hispanic	5.56%	6
Not Hispanic	19.44%	21
Haitian	23.15%	25
Other	51.85%	56

**Table 2:** Demographics for parents.

Characteristic	Percentage in Sample	n
Parents’ sex		
Male	9.26%	10
Female	90.74%	98
Parents’ race		
Black or African American	88.89%	96
White	2.78%	3
American Indian or Alaskan	1.85%	2
Multiracial	0.93%	1
Other	3.70%	4
Not reported	1.85%	2
Parents’ ethnicity		
Hispanic	4.63%	5
Not Hispanic	35.19%	38
Haitian	12.04%	13
Other	43.52%	47
Not reported	4.63%	5
Parents’ education		
Some high school	35.19%	38
High school graduate	28.70%	31
Some college or associate’s degree	22.22%	24
Bachelor’s degree	6.48%	7
Graduate degree	5.56%	6
Not reported	1.85%	2
Parents’ income		
USD 11,720–USD 35,743	87.98%	95
USD 35,744–USD 47,297	5.55%	6
USD 47,298–USD 75,000	1.85%	2
USD 75,000+	0.93%	1
Not reported	3.70%	4
Parents’ marital status		
Single/never married	35.19%	38
Married	10.19%	11
Living with a partner	4.63%	5
Separated/divorced	3.71%	4
Widowed	1.85%	2
Unknown	44.44%	48

**Table 3:** Descriptive statistics for variables pre-intervention.

Variables	Mean	SD	Min	Max	1	2	3	4	5
1. BASC-3	60.36	14.38	38	105	-	0.20 *	0.11	0.12	0.04
2. LAP-D FM	22.27	3.22	11	28		-	0.60 ***	0.50 ***	0.30 *
3. LAP-D FW	20.70	5.98	4	31			-	0.67 ***	0.35 **
4. BSRA-3	49.72	17.70	4	79				-	0.34 **
5. HTKS	12.34	15.07	0	57					-

Note. *** *p* < 0.001; ** *p* < 0.01; * *p* < 0.05.

**Table 4:** Descriptive statistics for variables post-intervention.

Variables	Mean	SD	Min	Max	1	2	3	4	5
1. BASC-3	51.85	11.99	33	83	-	0.13	0.09	0.18	0.17
2. LAP-D FM	23.28	2.67	16	28		-	0.69 ***	0.51 ***	0.38 ***
3. LAP-D FW	22.98	5.35	5	31			-	0.60 ***	0.47 ***
4. BSRA-3	63.82	14.56	8	83				-	0.47 ***
5. HTKS	21.75	17.63	0	58					-

Note. *** *p* < 0.001.

**Table 5:** Estimates of model regression coefficients with school readiness as the outcome, 95% confidence intervals of those coefficients, and *p*-values for pre-intervention data.

Variable	β	SE	95% CI	*p*
Pre-intervention model with HTKS as predictor				
HTKS scores	0.41	0.14	[0.13, 0.69]	0.004 **
Parent education	−0.20	1.35	[−2.90, 2.50]	0.88
Parent income	−0.77	0.89	[−2.56, 1.01]	0.39
Age	0.03	0.51	[−1.00, 1.07]	0.94
$R^{2}$	0.12			
Pre-intervention model with FM as predictor				
FM scores	2.10	0.47	[1.16, 3.04]	<0.001 ***
Parent education	0.12	0.81	[−1.48, 1.72]	0.88
Parent income	−0.14	0.45	[−1.03, 0.75]	0.76
Age	0.30	0.34	[−0.38, 0.98]	0.39
$R^{2}$	0.21			
Pre-intervention model with FW as predictor				
FW scores	2.00	0.25	[1.50, 2.51]	<0.001 ***
Parent education	0.69	0.93	[−1.16, 2.54]	0.46
Parent income	−0.85	0.53	[−1.90, 0.21]	0.11
Age	−0.11	0.36	[−0.82, 0.61]	0.76
$R^{2}$	0.40			
Pre-intervention model with all predictors				
FM scores	1.38	0.71	[−0.03, 2.79]	0.05
FW scores	1.78	0.40	[1.04, 2.52]	<0.001 ***
HTKS scores	0.11	0.12	[−0.13, 0.35]	0.38
Parent education	−0.22	1.08	[−2.38, 1.95]	0.84
Parent income	−1.67	0.78	[−3.23, −0.11]	0.03 *
Age	0.21	0.43	[−0.64, 1.07]	0.61
$R^{2}$	0.46			

Note. *** *p* < 0.001; ** *p* < 0.01; * *p* < 0.05.

**Table 6:** Estimates of regression coefficients with school readiness as the outcome, 95% confidence intervals of those coefficients, and *p*-values for post-intervention data.

Variables	β	SE	95% CI	*p*
Post-intervention model with HTKS as predictor				
HTKS scores	0.35	0.07	[0.20, 0.49]	<0.001 ***
Parent education	0.52	0.93	[−1.34, 2.38]	0.58
Parent income	−0.30	0.53	[−1.35, 0.76]	0.58
Age	0.37	0.33	[−0.33, 1.08]	0.29
$R^{2}$	0.25			
Post-intervention model with FM as predictor				
FM scores	2.09	0.49	[1.11, 3.07]	<0.001 ***
Parent education	0.40	0.83	[−1.25, 2.04]	0.63
Parent income	−0.31	0.46	[−1.23, 0.61]	0.51
Age	0.12	0.36	[−0.58, 0.84]	0.72
$R^{2}$	0.19			
Post-intervention model with FW as predictor				
FW scores	1.39	0.23	[0.91, 1.86]	0.001 ***
Parent education	0.22	0.76	[−1.29, 1.73]	0.77
Parent income	−0.66	0.44	[−1.54, 0.22]	0.14
Age	0.16	0.33	[−0.48, 0.81]	0.61
$R^{2}$	0.30			
Post-intervention model with all predictors				
FM scores	0.23	0.64	[−1.05, 1.51]	0.72
FW scores	0.99	0.36	[0.28, 1.70]	0.007 **
HTKS scores	0.19	0.08	[0.02, 0.33]	0.02 *
Parent education	0.22	0.93	[−1.64, 2.07]	0.81
Parent income	−0.69	0.56	[−1.81, 0.43]	0.22
Age	0.18	0.36	[−0.55, 0.92]	0.61
$R^{2}$	0.33			

Note. *** *p* < 0.001; ** *p* < 0.01; * *p* < 0.05.

## Data Availability

The datasets presented in this article are not readily available because the data are part of an ongoing study. Requests to access the datasets should be directed to KCH.

## Abbreviations

The following abbreviations are used in this manuscript:

EBP	Externalizing Behavior Problems
HTKS	Head–Toes–Knees–Shoulders
LAP-D	Learning Accomplishment Profile-Diagnostic
BASC-3	Behavior Assessment System for Children 3rd
BSRA-3	Bracken School Readiness Assessment 3rd Edition
